# 2528 Variable utilization of cross-sectional imaging prior to percutaneous peripheral vascular interventions

**DOI:** 10.1017/cts.2018.315

**Published:** 2018-11-21

**Authors:** Nathan K. Itoga, Kenneth Tran, Vivian Ho, Venita Chandra, Ronald L. Dalman, Edmund J. Harris, Jason T. Lee, Matthew W. Mell

**Affiliations:** 1 Stanford University School of Medicine; 2 Department of Surgery, Division of Vascular Surgery, Stanford University

## Abstract

OBJECTIVES/SPECIFIC AIMS: Reducing radiologic exams has been a focus of cost reduction in healthcare systems. The utility and justification of obtaining cross-sectional imaging (PPCSI) before surgical intervention continues to be evaluated. For peripheral artery disease (PAD) consensus guidelines regarding PPCSI do not exist and may be influenced by patient complexity, variation of disease presentation, and physician preference. The objective of this study was to determine the utility of PPCSI before percutaneous PAD intervention. METHODS/STUDY POPULATION: Patients receiving first-time endovascular revascularization procedure for PAD from 2013 to 2015 were evaluated for PPCSI done within 180 days prior to revascularization. Patient and physician demographics, perioperative characteristics, and disease distribution/severity were evaluated. The primary outcome was technical success defined as improving inflow and/or revascularization of the target outflow vessels to <50% stenosis. RESULTS/ANTICIPATED RESULTS: Of the 348 patients who underwent an attempted revascularization procedure 159 (45.7%) patients underwent PPCSI, including 151 CTA and 8 MRA. Of these, 48% were ordered by the referring provider (84% at an outside institution), and 52% were ordered by the treating physician. PPCSI was performed a median of 26 days (IQR 9-53) prior to procedure. Individual vascular surgeon practice identified PPCSI rates ranging from 31% to 70%. On multivariate analysis chronic kidney disease (OR=0.35; CI 0.17–0.73) had the strongest effect against of PPCSI, and Inpatient/ED evaluation (OR=3.20; CI 1.58–6.50), aorto-iliac (OR=2.78; CI 1.46–5.29) and femoral-popliteal occlusions (OR=2.51; CI 1.38–4.55) most strongly predicted PPCSI. After excluding 31 diagnostic procedures, technical success did not differ between endovascular procedures with PPSCI (91.3%) or without PPCSI (85.6%), *p*=0.11. When analyzing 89 femoral-popliteal occlusions, technical success was higher with PPCSI (88%) compared to procedures without PPSCI (69%), *p*=0.026. DISCUSSION/SIGNIFICANCE OF IMPACT: PPCSI use is influenced by inpatient status, chronic kidney disease, and anatomic consideration. PPCSI was not associated with overall technical success although it appeared beneficial for femoral-popliteal occlusions. Routine practices of ordering of PPCSI may not be warranted when considering technical success but may be important in treatment planning. Further studies are warranted to determine if radiation, cost, and contrast load justify PPCSI.

